# Impact of Virtual Learning on Mothers With Children in Elementary School: A Psychosocial Viewpoint

**DOI:** 10.7759/cureus.30257

**Published:** 2022-10-13

**Authors:** Mohammed A Aljaffer, Mohammed T Alzahrani, Ali E Shehadah

**Affiliations:** 1 Psychiatry, King Saud University, Riyadh, SAU; 2 Medicine, King Saud University Medical City, Riyadh, SAU; 3 Medical Student, King Saud University, Riyadh, SAU

**Keywords:** children, elementary school, mothers, stress, virtual learning

## Abstract

Background

Measuring the impact of virtual learning (VL), specifically psychosocially, and its consequences have been poorly studied because VL has never been implemented in this way before worldwide. To our knowledge, no studies in Saudi Arabia have addressed this topic, with very limited available literature internationally. This study aims to evaluate the psychosocial effects of VL on mothers of children in elementary school and its relation to psychosocial factors.

Methodology

Using an anonymous online questionnaire posted on social media, a quantitative, observational, cross-sectional study was conducted from May through December 2021 in Riyadh, Kingdom of Saudi Arabia. A total of 460 mothers consented to complete the study questionnaire. The questionnaire consisted of a socioeconomic section and collected information on perceived stress via the Perceived Stress Scale (PSS-14).

Results

The overall PSS-14 score showed a moderate stress level. Our results showed that as age groups tended to rise, stress scores tended to decline. Diabetes was a significant variable contributing to high stress. Verbal abuse toward a child essentially reflects an increase in stress. Mothers with familial conflicts were more prone to high stress.

Conclusions

The consequences of VL on mothers and the whole family are alarming. Stress, verbal and physical abuse, and unhealthy family dynamics are strongly associated with such a way of learning. The impact of emotional and behavioral changes among this group of individuals needs to be further investigated.

## Introduction

Coronavirus disease 2019 (COVID-19) emerged in Wuhan, China in December 2019 and is caused by severe acute respiratory syndrome coronavirus 2 (SARS-CoV-2) [1]. On March 11, 2020, the World Health Organization (WHO) declared it a pandemic [2]. Throughout 2020, the pandemic resulted in governments taking strict actions to protect their citizens and minimize the spread of infection. Nonetheless, the pandemic resulted in numerous unfortunate impacts globally, and health, economy, and education have all been affected. The regular educational process could not be continued to avoid the risk of spreading the virus.

On August 15, 2020, the Minister of Education from the Kingdom of Saudi Arabia, Dr. Hamad bin Mohammed Al Al-Sheikh, announced a mechanism for resuming the new school year 2020/2021 via virtual learning (VL) programs for seven weeks in all public schools nationwide [3]. Later, the Ministry announced an extension of the period to cover the entire academic year. Dr. Al-Sheikh also explained that this announcement was made after consulting with the Ministry of Health, the Ministry of Communications and Information Technology, the Education & Training Evaluation Commission, and the Human Capital Development Program [4].

During pandemics in general, people are known to experience psychological distress [5]. Studies have shown that lockdowns and school closures contribute to increased child abuse [6,7]. The pandemic led to increased anxiety, depression, and stress [8,9].

The objectives of this study are to evaluate the psychosocial effects of VL on mothers with elementary school children and estimate the prevalence of stress among mother mothers with elementary school children. Therefore, in our study, we hypothesize that VL negatively impacts mothers with elementary school children with an increased level of psychological stress.

## Materials and methods

Ethical considerations

Ethical approval for this study was provided by Health Sciences Colleges Research on Human Subjects/King Saud University, College of Medicine (approval number: 21/0383/IRB on April 22, 2021). All adult participants provided written informed consent to participate in this study. Confidentiality was assured by assigning each participant a code number for analysis. This study was performed in accordance with the Declaration of Helsinki. No financial aid or awards were given, nor was there any conflict of interest with the participants.

Study design

We conducted a quantitative, observational, cross-sectional study from May to December 2021 in Riyadh, Kingdom of Saudi Arabia. Data were collected through an online survey using a questionnaire distributed through social media platforms in May 2021. There was a risk of sampling bias which we tried our best to minimize by distributing an online questionnaire using a third party.

Inclusion and exclusion criteria

All participants were mothers of elementary school children and were between the ages of 18 to 60 years who spoke Arabic. Non-Arabic speakers or mothers who did not meet the age criteria were excluded.

Data collection

The following variables were included: age 18-60 years, occupation, educational level, marriage status, number of children, and socioeconomic status.

Mothers of elementary school children were the target population. The single proportion formula with a prevalence of 50% was used due to a lack of previous studies estimating the proportion of stress in mothers of elementary school children undergoing VL. The accuracy of the estimate was 5% and the confidence level was 95%. Using the single proportion formula the outcome was 384. The sample size was adjusted with 10% of the base of the sample to account for incomplete survey responses. Therefore, the sample size after adjustment was 423.

The self-reported questionnaires had two parts. The first part targeted the socioeconomic aspect, which gathered the following data about the participants: age, marital status, educational level, employment, monthly household income, number of children, number of children enrolled in elementary school at the time of the study, chronic illnesses, consumption of alcohol, smoking, illegal drug use, sleep habits, diet, exercise, presence of familial conflicts, child abuse, and the impact of VL on the mothers.

The second part of the questionnaire was the Perceived Stress Scale (PSS-14). It is a classic stress assessment tool that was originally developed in 1983 and has been considered a good choice for determining perceived stress. The questions in this scale relate to one’s feelings and thoughts during the last month. All candidates were asked to indicate how often they felt or thought a specific way. Each question was treated as a separate question, although there was some similarity.

The mean and standard deviation were used to describe the continuous metric variables. The frequency and percentage analysis were applied for categorically measured variables such as the participants’ educational level and occupation. The histogram and the Kolmogorov-Smirnov test were used to assess the statistical normality assumption of metric variables.

Statistical analysis

The chi-square test of independence (χ^2^) was used to evaluate the association between mothers’ risk of having high stress with their categorically measured socioeconomic characteristics and their health and habits. Furthermore, a continuity-adjusted Yates (χ^2^) test and likelihood ratio (LR)-adjusted chi-square test values and associated p-values were quoted when the assumptions of the chi-square test for expected counts were violated for the 2 × 2 and bigger contingency tables, respectively. The author’s scoring manual of the PSS-14 questionnaire was used to compute the mother’s total PSS and its subdomain concepts (distress and difficulty coping) by adding the items that comprised each item after reverse coding the positively worded statements to yield scores that denoted more stress as the scores increased. The reliability of the PSS-14 questionnaire was assessed using Cronbach’s alpha test. The multiple-response dichotomy analysis was applied to the questions and measured with more than one option. The mother’s total PSS was dichotomized based on the median value of the sample into low- and high-perceived stress (median PSS-14 score, 26 points). The multivariate logistic binary regression analysis assessed the combined and individual associations between the mother’s socioeconomic characteristics, such as habits and parenting practices, and other relevant factors with their odds of having had high perceived stress during the VL of their children associated with the COVID-19 pandemic restrictions. The association between the tested predictor independent variables with the mother’s odds of having been highly stressed due to children’s VL was expressed as an odds ratio (OR) with a 95% confidence interval (95% CI). SPSS version 21 (IBM Corp., Armonk, NY) was used for the statistical analysis, and the p-value was considered statistically significant at an alpha level of 0.05.

## Results

We collected 460 responses from mothers of elementary school children in the KSA. Overall, 6.7% of the mothers were aged 18-30 years, 34.1% were aged 31-40 years, 50.7% were aged 41-50 years, and the remaining 8.5% were aged 51-60 years. Further, the survey revealed that 94.6% of participants were currently married, and the rest 5.4% were single. Additionally, 5.4% of the mothers had an elementary educational level, 3.9% had completed their intermediate educational level, 14.1% had completed their high school level, and the majority of the mothers (76.5%) had a university degree.

Moreover, 45.7% of the mothers were unemployed housewives and 54.3% were employed. The monthly income of their households was as follows: 9.6% had a household income of <5,000 SAR per month, 19.8% had an income of 5,000-10.000 SAR per month, 30.9% had an income of 10,001-15,000 SAR per month, 23.9% had an income of 15,001-20,000 SAR per month, and the remaining 15.9% of the households had a monthly income of >20,000 SAR (Table 1).

**Table 1:** Mothers’ socioeconomic characteristics.

	Frequency	Percentage
Age group		
18–30 years	31	6.7
31–40 years	157	34.1
41–50 years	233	50.7
51–60 years	39	8.5
Marital state/social state		
Ever married (divorced/separated/widow)	25	5.4
Married	435	94.6
Educational level		
Elementary	25	5.4
Intermediate	18	3.9
Secondary	65	14.1
University	352	76.5
Employment		
No	210	45.7
Yes	250	54.3
Monthly household income		
<5,000 SAR	44	9.6
5,000–10,000 SAR	91	19.8
10,001–15,000 SAR	142	30.9
15,001–20,000 SAR	110	23.9
>20,000 SAR	73	15.9
Number of children		
1	32	7
2	56	12.2
3	72	15.7
4	101	22
>4 children	199	43.3
Number of children in elementary level		
1	284	61.7
2	139	30.2
3	24	5.2
4	13	2.8

The mothers’ general health characteristics during their children’s VL showed that 22.20% of the mothers had a chronic illness (Table 2). Most mothers (40.4%) had been diagnosed with diabetes, 27.3% with hypertension, 20.2% with hypothyroidism, 12.1% with a respiratory disease like asthma, and 21.2% had chronic illnesses. When they were asked to indicate whether they consumed cigarettes, alcohol, or cannabis, most mothers stated that they did not consume any of these substances, except for 10 mothers, who stated they were smokers, while one mother stated that she consumed alcohol. The findings also showed that 51.7% of the mothers experienced sleeping difficulties. When they were asked to indicate the number of hours they slept per night, 3.9% slept for ≤three hours per night, 58.3% slept between four and six hours per night, 36.1% slept between seven and nine hours per night, and 1.7% slept between 10 and 12 hours per night (Table 2).

**Table 2:** Descriptive analysis of mothers’ health and exercise as well as practices during their children’s VL.

Question/Answer	Frequency	Percentage
Do you have any chronic illnesses?		
No	358	77.8
Yes	102	22.2
Type of chronic illness:		
Diabetes	40	40.4
Hypertension	27	27.3
Hypothyroidism	20	20.2
Respiratory asthma	12	12.1
Other	21	21.2
Do you consume any of the following: cigarette smoking, alcohol, or cannabis?		
No	450	97.8
Yes	10	2.2
Do you experience any difficulty sleeping?		
No	222	48.3
Yes	238	51.7
How many hours do you sleep per night?		
≤3 hours/day	18	3.9
4–6 hours/day	268	58.3
7–9 hours/day	166	36.1
10–12 hours	8	1.7
How often do you need to take sleeping pills to aid your sleep?		
Never	399	86.7
Rarely	25	5.4
Sometimes	32	7
Often	4	0.9
Do you follow a balanced and healthy diet style?		
No	219	47.6
Yes	241	52.4
How often do you exercise?		
Never	84	18.3
Rarely	120	26.1
Sometimes	218	47.4
Often	38	8.3
Have any familial conflicts occurred due to the pressure caused by virtual learning?		
No	253	55
Yes	207	45
Type of conflicts encountered:		
Minor familial conflicts	182	82
Physical abuse	7	3.2
Verbal abuse	25	11.3
Emotional abuse	50	22.5
How has your children’s virtual learning affected you?		
Negatively	181	39.3
No impact	192	41.7
Positively	87	18.9
Are your children being treated abusively?		
No	367	79.8
Yes	93	20.2
Types of abuse used with children during VL		
Verbal	65	69.9
Physical	7	7.5
Emotional	30	32.3

Participants were asked to rate their need to take sleeping pills to aid their sleep using a Likert-type scale, and the results showed that 86.70% of the mothers had never used sleeping pills, 5.4% rarely used sleeping pills, 7% sometimes consumed sleeping pills, and 0.9% consumed pills frequently. Moreover, 52.4% of the mothers followed a healthy diet but 47.6% did not. The mothers were also asked to self-rate their exercise level on a Likert-type scale. Overall, 18.3% had never exercised, 26.1% rarely exercised, 47.4% sometimes exercised, and 8.3% often exercised. Additionally, 45% of the mothers admitted experiencing familial conflicts caused by their children’s VL stress. Those who had experienced family conflicts were asked to mention the type of conflicts they faced. The results revealed that 82% of such mothers had experienced minor family conflicts, 3.2% had experienced physical abuse, 11.3% experienced verbal abuse, and 22.5% had experienced emotional abuse (Table 2).

The mothers were asked to indicate with No/Yes whether they thought their children were abused at home during the pandemic-associated VL. The findings showed that 20.2% of the mothers agreed that their child was treated abusively. These women were asked to state the type of abuse their child was exposed to. Results demonstrated that 96.9% of the mothers reported verbal abuse, 7.5% reported physical abuse, and 32.3% reported emotional abuse (Table 2).

PSS-14

The PSS scale was divided into coping and distress-related questions (Table 3). The mothers’ top perceived coping aspect was their extent of thinking about the things they had to accomplish which had received a mean rating of 2.97/4, suggesting that those mothers may have been under pressure for the tasks they needed to fulfill while assisting their children’s VL. The mothers’ second top perceived coping indicator was their self-confidence in handling personal problems which received a mean rating of 2.68/4, indicating that those mothers may have a substantive sense of confidence in their ability to tackle their personal problems.

**Table 3:** Descriptive analysis of mothers’ perceptions of the indicators of distress and coping during the virtual learning of their children.

D = Distress, C = Coping		Mean	SD	Mean rank
Coping				
C1	In the last month, how often have you dealt successfully with irritating life?*	2.42	1.05	5
C2	In the last month, how often have you felt that you were effectively coping with important changes that were occurring in your life?*	2.49	1.09	4
C3	In the last month, how often have you felt confident about your ability to handle your personal problems?	2.68	1.04	2
C4	In the last month, how often have you felt that things were going your way?	2.36	1	8
C5	In the last month, how often have you been able to control irritations in your life?	2.37	1.03	6
C6	In the last month, how often have you felt that you were on top of things?	2.52	1.02	3
C7	In the last month, how often have you found yourself thinking about things that you have to accomplish?	2.97	0.98	1
C8	In the last month, how often have you been able to control the way you spend your time?	2.37	1.04	7
Distress				
D1	In the last month, how often have you been upset because of something that happened unexpectedly?	1.68	1.1	6
D2	In the last month, how often have you felt that you were unable to control important things in your life?	1.69	1.13	5
D3	In the last month, how often have you felt nervous and “stressed”?	2.42	1.09	1
D4	In the last month, how often have you found that you could not cope with all the things that you had to do?	2.05	1.04	3
D5	In the last month, how often have you been angered because of things that happened that were outside of your control?	2.24	0.97	2
D6	In the last month, how often have you felt difficulties were piling up so high that you could not overcome them?	1.91	1.16	4

The mothers’ overall PSS associated with their children’s VL was 25.72 points out of a maximum of 56 points (±7.21 points). When expressed as a percentage, the mothers’ overall score would be equivalent to 100 (25.75/56) = 45.9% perceived stress. This suggests general moderate perceived stress by mothers (Table 4).

**Table 4:** Descriptive analysis of mothers’ perceptions of distress, coping, and the overall PSS-14 scores. PSS-14: Perceived Stress Scale-14

PSS	Mean score	SD	Maximum possible score
Overall perceived stress scale	25.75	7.21	0–56 points
Perceived distress score	11.98	4.69	0–24 points
Perceived coping difficulties score	13.77	4.84	0–32 points

To better understand the reason why the mothers experienced higher or lower stress during their children’s VL period, bivariate and multivariate analyses were performed. The association between the mothers’ socioeconomic characteristics, perceptions, health outcomes, and psychological burden with their dichotomized PSS-14 (low/high) scores was evaluated. The findings from the bivariate analysis, as shown in Table 5, showed that the mother’s age group was correlated significantly with their perceived stress from their children’s VL. Women aged 31-40 years were found to be significantly more inclined to high stress from their children’s VL compared to other women in different age groups (p = 0.021), according to the chi-square test of independence. However, the analysis showed that the mothers’ current marital status, their educational attainment level, their employment status, as well as their household monthly income, the number of children, and the number of children at the elementary level, showed no significant correlation with their perceived stress while supervising their children who learned via virtual methods during the pandemic (Table 5).

**Table 5:** Bivariate analysis of mothers’ perceived stress during the virtual learning of their children.

Perceived stress				
	Low, n = 233	High, n = 227	Test statistic	P-value
Age group				
18–30 years	12 (5.2)	19 (8.4)	χ^2^ (3) = 9.69	0.021
31–40 years	69 (29.6)	88 (38.8)		
41–50 years	126 (54.1)	107 (47.1)		
51–60 years	26 (11.1)	13 (5.7)		
Marital state/social state				
Ever married (divorced/separated/widow)	10 (4.3)	15 (6.6)	χ^2^ (1) = 1.20	0.273
Married	223 (95.7)	212 (93.4)		
Educational level?				
Elementary	13 (5.6)	12 (5.3)	χ^2^ (3) = 0.58	0.901
Intermediate	8 (3.4)	10 (4.4)		
Secondary	35 (15)	30 (13.2)		
University	177 (76)	175 (77.1)		
Employment				
No	104 (44.6)	106 (46.7)	χ^2^ (1) = 0.197	0.657
Yes	129 (55.4)	121 (53.3)		
Monthly household income				
<5,000 SAR	23 (9.9)	21 (9.3)	χ^2^ (4) = 1.13	0.889
5,000–10,000 SAR	43 (18.5)	48 (21.1)		
10,001–15,000 SAR	70 (30)	72 (31.7)		
15,0001–20,000 SAR	57 (24.5)	53 (23.3)		
>20,000 SAR	40 (17.2)	33 (14.5)		
Number of children				
1	16 (6.9)	16 (7)	χ^2^ (4) = 0.7	0.951
2	30 (12.9)	26 (11.5)		
3	36 (15.5)	36 (15.9)		
4	48 (20.6)	53 (23.3)		
>4	103 (44.2)	96 (423)		
Number of children in elementary level				
1 child	152 (65.2)	132 (58.1)	χ^2^ (3) = 3.04	0.385
2 children	66 (28.3)	73 (32.2)		
3 children	10 (4.3)	14 (5.2)		
4 children	5 )2.1)	8 (3.5)		
Do you have any chronic illnesses?				
No	184 (79)	174 (76.7)	χ^2^ (1) = 0.40	0.550
Yes	49 (21)	53 (23.3)		
Type of chronic illness				
Diabetes	15 (6.4)	25 (11)	χ^2^ (1) = 3.03	0.082
Hypertension	19(8.2)	8 (3.5)	χ^2^ (1) = 4.460	0.035
Hypothyroid	11 (4.7)	9 (4)	χ^2^ (1) = 0.16	0.691
Respiratory asthma	6 (2.6)	6 (2.6)	χ^2^ (1) = 0.002	0.963
Other	8 (3.4)	13 (5.7)	χ^2^ (1) = 1.38	0.239
Substance use/abuse				
No	225 (96.6)	225 (99.1)	χ^2^ (1) = 2.421	0.119
Yes	8 (3.4)	2 (0.9)		
Do you experience difficulty sleeping?				
No	148 (63.6)	74 (32.6)	χ^2^ (1) = 44.02	<0.001
Yes	85 (36.5)	153 (67.4)		
How many hours do you sleep per night?				
≤3 hours/day	5 (.1	13 (5.7)	χ^2^ (3) = 24.11	<0.001
10-12 hours/day	1 (0.4)	7 (3.1)		
4-6 hours/day	121 (51.9)	147 (64.8)		
7-9 hours/day	106 (45.5)	60 (26.4)		
How often do you need to take sleeping pills to aid your sleep?				
Never	210 (90.1)	189 (83.3)	χ^2^ (3) = 7.41	0.060
Rarely	12 (5.2)	13 (5.7)		
Sometimes	9 (3.9)	23 (10.1)		
Often	2 (0.9)	2 (0.9)		
Do you follow a balanced and healthy diet style?				
No	87 (37.3)	132 (58.1)	χ^2^ (1) = 19.96	<0.001
Yes	146 (62.7)	95 (41.9)		
How often do you exercise?				
Never	41 (17.6)	43 (18.9)	χ^2^ (3) = 6.89	0.078
Rarely	50 (21.5)	70 (30.80		
Sometimes	119 (51.1)	99 (43.60		
Often	23 (9.9)	15 (6.6)		
Have you encountered familial conflicts due to pressures caused by virtual learning of your child?				
No	160 (68.7)	93 (41)	χ^2^ (1) = 35.65	<0.001
Yes	73 (31.3)	134 (59)		
Type of conflicts encountered				
Minor family conflicts	67 (28.8)	115 (50.7)	χ^2^ (1) = 23.1	<0.001
Physical abuse	1 (0.4)	6 (2.6)	χ^2^ (1) = 2.43	0.119
Verbal abuse	1 (0.4)	24 (10.6)	χ^2^ (1) = 23.02	<0.001
Emotional abuse	13 (5.6)	37 (16.3)	χ^2^ (1) = 13.6	<0.001
How has your children’s virtual learning affected you?				
Negatively	69 (29.6)	112 (49.3)	χ^2^ (2) = 22.88	<0.001
No impact	105 (45.1)	87 (38.3)		
Positively	59 (25.3)	28 (12.3)		
Are your children being treated abusively?				
No	202 (86.7)	165 (72.7)	χ^2^ (1) = 13.99	<0.001
Yes	31 (13.3)	62 (27.3)		
Type of abuse used with children during VL				
Verbal	22 (9.4)	43 (18.9)	χ^2^ (1) = 8.50	0.003
Physical	2 (0.9)	5 (2.2)	χ^2^ (1) = 0.64	0.426
Emotional	10 (4.3)	20 (8.8)	χ^2^ (1) = 3.85	0.050

In addition, this study showed that mothers’ history of having chronic illness did not correlate significantly with their perceived stress level (p = 0.550), though mothers known to have had diabetes were found to be slightly more predisposed to have high stress compared to non-diabetics (p = 0.085). Hypertensive mothers were found to be significantly less predicted to have high stress compared to non-hypertensive mothers (p = 0.035). The chronic conditions of the others did not correlate significantly with their perceived VL-associated stress, nor did their history of substance use/abuse correlate significantly with the stress associated with their children’s VL (Table 5).

The findings also showed that mothers known to have sleeping difficulties during the pandemic were significantly more inclined to high stress associated with their children’s VL than those who had no sleeping problems (p < 0.001), according to the chi-square test of associations. Additionally, mothers’ night sleep hour levels converged significantly on their perceived stress; mothers who slept between seven and nine hours per night were found to be considerably less inclined to develop high stress than those who generally slept <seven hours (p < 0.001), according to an LR-adjusted chi-square test of association. The mothers’ use of sleeping pills did not converge significantly on their perceived stress from their children’s VL. Mothers who followed a healthy diet plan had significantly less predisposition to have high stress from their children’s VL compared to those who did not follow a healthy diet plan (p < 0.001). The mothers’ exercise level did not correlate significantly with their perceived stress from VL (p = 0.078). Furthermore, mothers who experienced family problems associated with stress caused by their children’s VL were significantly more inclined to have had significant stress levels compared to those who did not experience such family issues (p < 0.001), as revealed by the chi-square test (Table 5).

The findings showed that mothers who experienced minor family conflicts were found to be significantly more stressed from their children’s VL compared to those who did not have minor family conflicts (p < 0.001). The mothers’ exposure to physical abuse did not converge significantly on their perceived stress (p = 0.119). Conversely, Mothers exposed to verbal abuse and emotional abuse were found to be significantly more predicted to have high stress from VL compared to those who experienced no such conflicts (p < 0.001). The mother’s perception of the impact of VL on their children showed a remarkable correlation with their perceived stress. Mothers who believed that VL had impacted them negatively perceived significantly greater stress compared to those who believed the children’s VL did not impact them or even had been positively impacted by their children’s new VL experience (p < 0.001) (Table 5).

**Figure 1:**
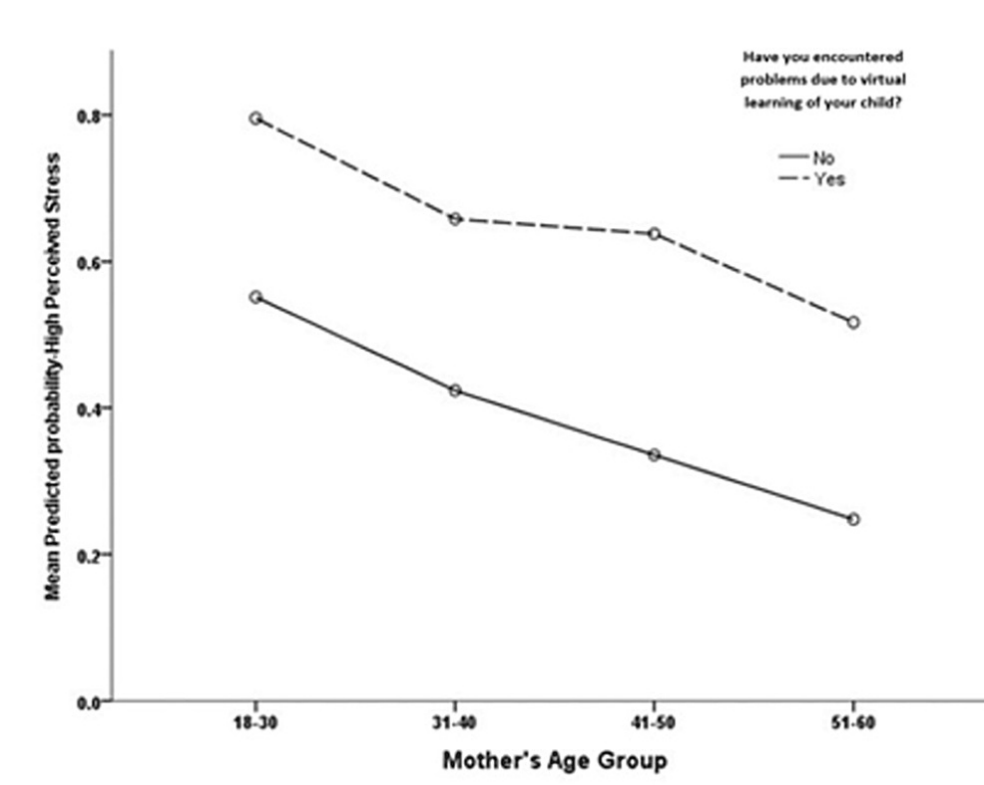
Association between mothers’ age group with their model predicted probability of having high perceived stress with subgroup analysis for parental experienced problems.

To ascertain the yielded findings from the bivariate analysis, the multivariate logistic binary regression analysis was used and mothers’ perceived stress level against the predictors of stress was regressed. The yielded multivariate analysis model (Table 6) showed that the mother’s age correlated significantly and negatively with their odds of having high VL-associated stress (p < 0.001), accounting for other predictor variables in the analysis. Figure 1 shows that as the mother’s age increased, their mean model-predicted probability of having a high PSS-14 score tended to decline, regardless of their having experienced conflicts during the VL of their children during the pandemic restrictions/lockdown in Saudi Arabia. The multivariate analysis showed that the mothers’ number of children and the number of children at the elementary level did not correlate significantly with their odds of having high stress from their children’s VL. Mothers who had experienced minor family problems were significantly more likely (3.27 times more) to have had higher stress levels than those with no minor family conflicts (p = 0.016). Mothers who reported the use of verbal abuse with their children during VL were found to be significantly more likely (18.78 times more) to have had high stress compared to mothers who did not report verbally abusing their children (p = 0.007). The children’s exposure to physical abuse did not converge significantly with the mothers’ stress levels (p = 0.059). Mothers who witnessed physical abuse of their children during VL were found to be slightly more likely to have had a high PSS-14 score compared to other mothers who had not witnessed such abuse (Table 6).

**Table 6:** Multivariate logistic binary regression analysis of the odds of the mothers having high stress during the virtual learning of their children (N = 460). PSS-14: Perceived Stress Scale-14

Variable	Multivariate adjusted odds ratio	95% CI for OR		P-value
		Lower	Upper	
Age group	0.681	0.503	0.922	0.013
Number of children	1.398	0.646	3.024	0.395
Problems experienced due to virtual learning	0.572	0.208	1.570	0.278
Had minor family problems	3.273	1.245	8.604	0.016
Use of verbal abuse with child	18.784	2.233	158.019	0.007
Use of emotional abuse with child	2.714	0.961	7.660	0.059
Difficulty sleeping	2.175	1.353	3.494	0.001
Amount of sleeping hours per night	0.746	0.526	1.058	0.100
Diabetes mellitus	3.526	1.529	8.132	0.003
Hypertension	0.394	0.146	1.064	0.066
Perceived impact of virtual learning on mother	0.795	0.581	1.087	0.150
Smoker	0.108	0.016	0.717	0.021
Follows healthy diet	0.474	0.308	0.730	0.001
Level of exercise self-rating	0.859	0.672	1.097	0.224
Constant	9.614			0.011
Dependent variables: PSS-14 score >26 points (No/Yes)				

Outcome data

The multivariate analysis revealed that mothers who experienced difficulty in sleeping were found to be at significantly increased odds (2.18 times higher) of high perceived stress compared to those who had no sleeping problems on average (p = 0.001). The number of hours for which each mother slept did not correlate significantly with their odds of having high VL-associated perceived stress levels (p = 0.100). Diabetic mothers were significantly more predicted (3.53 times more) to score higher on PSS-14 compared to non-diabetic mothers on average (p = 0.003). However, the mothers’ medical history of hypertension and other medical chronic illnesses did not converge significantly on their odds of having high stress during their children’s VL. The perceived impact of VL on the children according to the mothers did not correlate significantly statistically with the mother’s perceived stress. Those who smoked were found to be significantly less likely to have high stress (0.108 times less) compared to those who did not smoke (p = 0.021). Mothers who followed a healthy diet plan were found to be significantly less (0.474 times less) inclined to experience high stress from their children’s VL compared to those who did not follow a healthy diet plan (p = 0.001). The multivariate analysis showed that the mothers’ level of exercise did not correlate significantly with their perceived stress (p = 0.224) as shown in Table 6.

## Discussion

VL is a novel approach initiated during the COVID-19 pandemic. As there was no precedent, it was mandated to conduct research to assess the effects of this new educational tool. We assumed that it would have a negative impact on mothers of elementary school-going children and would increase stress and affect them negatively.

Socioeconomic part

As Åkerstedt et al. demonstrate, stress affects both the quality as well as the duration of sleep; therefore, in stress-inducing situations, people tend to have sleep disturbances. The results of our study suggest the same; 51.7% of the participants experienced difficulties in sleeping [10].

Furthermore, when mothers were asked about child abuse, 20.2% of children were abused verbally and emotionally, and 7.5% were abused physically. This shows the increased abuse rate during stress-inducing situations, such as lockdowns and school closures. This supports the findings of a previous study by Brown et al. where higher stress was associated with more potential for child abuse [11].

PSS-14

The mothers averaged a score of 25.72 out of 56 on the PSS-14, with 7.21 as the standard deviation. We can see the high variation in our selected population, indicating a moderate level of stress, in general.

As Weisberg et al. stated that 50% of generalized anxiety disorders have an onset between ae 20 and 47, we also found that age is an important factor in determining stress experienced by mothers, as mothers in the 31-40-year age group were more prone to developing stress than other age groups in our study. Using multivariate regression analysis, we noted that age is highly significant as mothers’ age group tended to rise but PSS-14 scores tended to decline [12].

Diabetic participants were more prone to high stress. Multivariate analysis showed that diabetes was significantly associated; diabetic mothers’ stress levels were 3.53 times higher than non-diabetic mothers. Diabetes increases stress levels. As demonstrated by DeJean et al., anxiety, psychological stress, and diabetes aggravate one another [13]. Stress levels also tended to be higher in mothers with difficulty sleeping. Our study showed that the stress levels of mothers with sleeping difficulties were 2.18 times more in comparison to mothers with trouble-free sleep. This is in accordance with the study by Åkerstedt et al. which showed an association of lower sleeping hours with higher stress levels [10].

Mothers who followed a healthy diet were found to be less stressed compared to mothers who did not. This highlights the importance of a healthy lifestyle, which was also demonstrated by El Ansari et al. who conducted a study on university students in the United Kingdom and reported that students who consumed a healthy diet were significantly less stressed than their colleagues who followed an unhealthy diet [14]. Furthermore, findings showed that mothers who experienced familial conflicts caused by their children’s VL had 3.27 times higher stress levels than those who did not report any familial conflicts. Moreover, mothers who were verbally and emotionally abused during the VL of their children experienced high stress compared to non-abused mothers. Rowe et al. reported the harmful effects of verbal abuse on nurses which led to higher stress levels, decreased job satisfaction, and more missed workdays [15]. Another notable piece of literature is the study by Kind et al. on professional caregivers in youth institutes in Switzerland which reported higher stress levels along with burnout symptoms in caregivers who reported verbal abuse compared to those who did not. The aforementioned studies emphasize the adverse effects verbal abuse can have on stress levels, which is consistent with the results of our study [16].

The way mothers perceived the impact of VL on their life had a direct impact on their stress levels. Mothers who believed that VL of their children had a negative impact on their life reported higher stress levels than those who did not report such an impact. This is consistent with the study by Song et al. who implied in their study on adolescents in Korea the negative impacts pessimism has on increased COVID-19-related stress, while, on the other hand, optimism showed a decreased level of stress [17]. Moreover, Arslan et al. studied the effects of pessimism and optimism on COVID-19-related stress in Turkish adults. The study showed that pessimistic individuals are more likely to report higher stress levels and low self-esteem when compared to optimistic individuals. This highlights the enormous effect of one’s perception and the psychosomatic effect of stress and pessimistic vision [18].

Among the limitations of this study was the fact that the questionnaire was subjectively causing a response bias; for instance, what one mother would consider as a form of verbal abuse another would not. An online survey, in general, has limitations in assessing participant responses in contrast to face-to-face interviews. Further, abuse patterns before the pandemic were not assessed, which did not allow for a proper comparison. Stress in mothers was also evaluated late in the school year, which could lead to recall bias. Finally, the age of the children undergoing VL was not considered.

We recommend that awareness programs should be conducted particularly aimed at mothers regarding difficulties encountered due to VL. Further, the Ministry of Education should provide tools that can minimize the stress encountered by mothers. Providing hotlines and professional consultation tools may also significantly help improve this valuable way of learning. Lastly, future studies should be conducted to assess the delayed consequences of VL.

## Conclusions

VL was introduced as a measure to ensure the continuation of the education process amid the COVID-19 pandemic, requiring parents, especially mothers, to be more actively involved in their children’s education. Our study aimed to study the effects of such a new burden on the mother’s psychological health. We concluded that young age, unhealthy diet, difficulty sleeping, and diabetes were among the leading factors in increasing stress levels in mothers.

The consequences of VL on mothers and the whole family were quite alarming. Stress, verbal and physical abuse, and unhealthy family dynamics were strongly associated with the potential impacts of VL. The impact of emotional and behavioral changes among this group of individuals needs to be further addressed and investigated.
